# The Publication of the Transactions of the American Medical Association

**Published:** 1882-07

**Authors:** 


### EDITORIAL DEPARTMENT.

The Publication of the Transactions of the American Medical Association.—At the late meeting of the American Medical Association, the committee which had been appointed last year to consider the propriety of publishing the transactions of the Association in a weekly journal, in a manner similar to those of the British Medical Association, reported a series of resolutions in favor of such action. The resolutions state that the interests of the Association would be promoted by the publication of its transactions in such a manner, instead of in the bulky annual volume heretofore issued. There can be no question of the increased value of the transactions if published and placed before the profession in a reasonable time, and in an accessible shape. A board of nine trustees was appointed, whose duty it will be to agree upon a plan of a "Journal of the American Medical Association," and to secure sufficient pledges of support, by sending circulars explaining such plan, as will render the journal self-sustaining. It is the further duty of this board of trustees to "agree upon the best method of publishing such journal, the best editorial services it can secure to take charge of the work, and the best plan of its issue." It shall also, under all circumstances, retain entire control over the advertising, as well as all other pages of the journal. The editor is to receive a salary of six thousand dollars per year, out of which he is to pay the services of his assistants, while the income from the subscriptions and advertisements is to go into the treasury of the Association. While we have no doubt of the great advantage of publishing the transactions in this manner, nevertheless the success of the new journal, should it be established, will depend very largely upon the qualifications of the editor. Undoubtedly there are gentlemen with experience in the conduct of medical journals in this country who would be as well qualified for the position as Mr. Ernest Hart has shown himself to be in a similar position in England. Yet the Board of Trustees will have a duty requiring great tact and judgment in the selection of an editor.

The Board of Trustees consists of Drs. Davis, of Chicago; Moore, of New York; Toner, of Washington; Campbell, of Georgia; Packard, of Pennsylvania; Connor, of Michigan; Hooper, of Arkansas; Garcelon, of Maine, and McMurtry, of Kentucky. The board is on the whole a good one, though only two of the members are practically conversant with the duties of a medical journalist.

